# Ultrasensitive proteomic quantitation of cellular signaling by digitized nanoparticle-protein counting

**DOI:** 10.1038/srep28163

**Published:** 2016-06-20

**Authors:** Thomas Jacob, Anupriya Agarwal, Damien Ramunno-Johnson, Thomas O’Hare, Mehmet Gönen, Jeffrey W. Tyner, Brian J. Druker, Tania Q. Vu

**Affiliations:** 1 Department of Biomedical Engineering, Oregon Health & Science University, Portland OR 97201, USA; 2 OHSU Center for Spatial Systems Biomedicine, Oregon Health & Science University, Portland OR 97201, USA; 3 Division of Hematology and Medical Oncology, The Knight Cancer Institute, Oregon Health & Science University, Portland OR 97239, USA; 4 Division of Hematology and Hematologic Malignancies, Huntsman Cancer Institute, University of Utah, 2000 Circle of Hope, Salt Lake City, Utah 84112, USA; 5 Department of Industrial Engineering, Koc University, Istanbul, Turkey; 6 Department of Cell Developmental & Cancer Biology, Oregon Health & Science University, Portland OR 97239, USA.; 7 Howard Hughes Medical Institute, Portland, Oregon, USA.

## Abstract

Many important signaling and regulatory proteins are expressed at low abundance and are difficult to measure in single cells. We report a molecular imaging approach to quantitate protein levels by digitized, discrete counting of nanoparticle-tagged proteins. Digitized protein counting provides ultrasensitive molecular detection of proteins in single cells that surpasses conventional methods of quantitating total diffuse fluorescence, and offers a substantial improvement in protein quantitation. We implement this digitized proteomic approach in an integrated imaging platform, the single cell-quantum dot platform (SC-QDP), to execute sensitive single cell phosphoquantitation in response to multiple drug treatment conditions and using limited primary patient material. The SC-QDP: 1) identified pAKT and pERK phospho-heterogeneity and insensitivity in individual leukemia cells treated with a multi-drug panel of FDA-approved kinase inhibitors, and 2) revealed subpopulations of drug-insensitive CD34+ stem cells with high pCRKL and pSTAT5 signaling in chronic myeloid leukemia patient blood samples. This ultrasensitive digitized protein detection approach is valuable for uncovering subtle but important differences in signaling, drug insensitivity, and other key cellular processes amongst single cells.

Many important proteins, including signaling and regulatory proteins, are present at low copy number and therefore difficult to detect and quantitate in individual cells^1^,^2^. Protein phosphorylation, for example, underlies ubiquitous and vital signaling processes; however, phosphoactivated proteins exist at extremely low abundance in single cells^3^,^4^,^5^. Moreover, many therapeutic compounds, such as kinase inhibitors, target and suppress protein signaling^6^,^7^,^8^,^9^,^10^,^11^, further decreasing endogenous levels of signaling molecules, and posing additional challenges to detecting signaling molecules in single cells. Individual cells in a population are believed to contain differing levels of signaling molecules. Such cellular heterogeneity may hold important keys to understanding the degree of effectiveness of some therapeutic treatments^12^,^13^,^14^,^15^,^16^, as well as understanding important cell biological mechanisms (e.g. cellular proliferation and disease recurrence^17^,^18^,^19^,^20^,^21^) but may be challenging to detect. Tools that provide increased sensitivity in quantitative detection of low abundant proteins in individual cells would provide important, detailed information on subtle cellular differences that otherwise may be overlooked^14^.

A technical challenge in measuring low abundance proteins is attaining sufficient sensitivity necessary to reliably detect and quantify levels of proteins above background noise. We introduce a molecular imaging approach to quantify proteins of low abundance by counting discrete fluorescence-tagged proteins. This digitized protein quantification method is implemented within an integrated platform, the single cell quantum-dot platform (SC-QDP), which uses quantum dots (QDs) as the fluorescent reporter, by which to count discrete protein complexes. QD are intensely bright, bleaching-resistant semiconductor nanoparticles that have matured as valuable probes for multi-color immunofluorescence and for tracking the dynamics of single molecules^22^,^23^ yet, the advantages of digitized proteomic quantification using QDs, or other dyes have not been fully recognized. The SC-QDP also has very high cell retention, enabling assays of limited quantities of cell sample, thereby overcoming a major bottleneck in assay of primary patient material. We demonstrate that the SC-QDP quantitates phosphoresponse heterogeneity in human acute myeloid leukemia MOLM14 cells to kinase inhibitor drugs (KIs) and identifies KI-insensitive CD34+ cells in patients diagnosed with chronic myeloid leukemia. The molecular sensitivity offered by this digitized proteomic approach is valuable for revealing differences in signaling and other important cellular processes in single cells that are otherwise challenging to quantitate.

## Results

### Single cell quantum-dot platform (SC-QDP)

**Figure 1:**
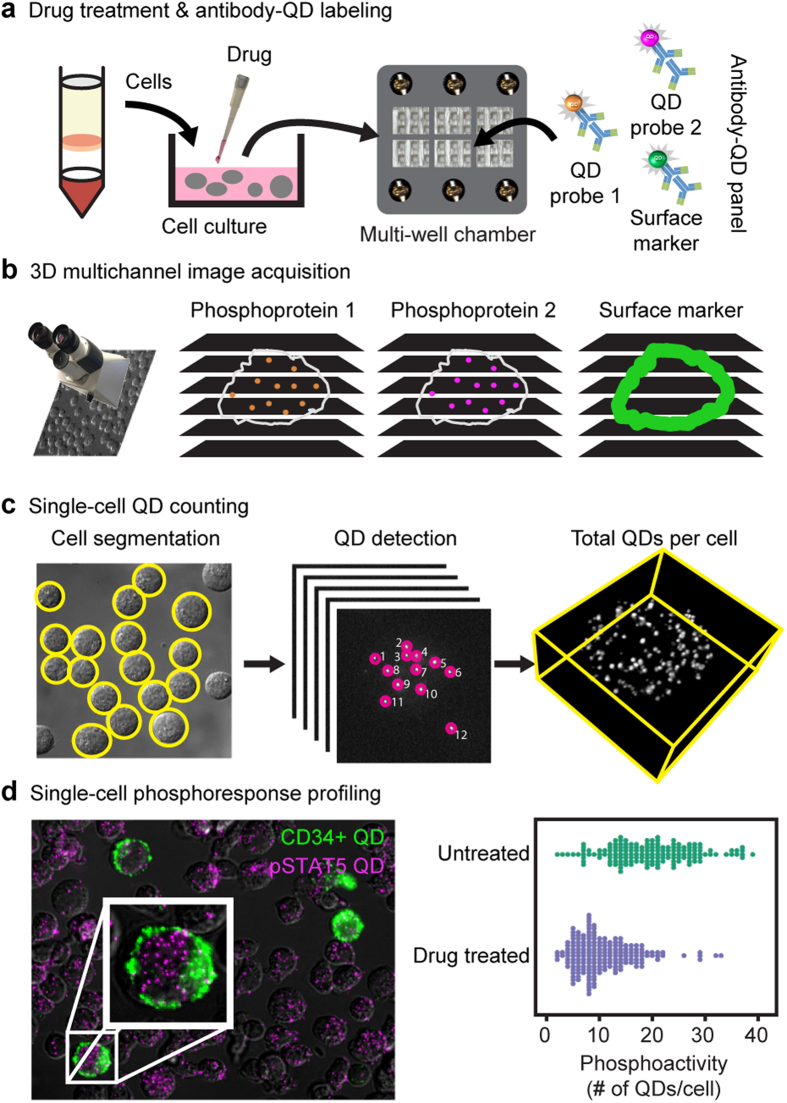
Digitized phosphoprotein quantitation by the single cell quantum-dot platform. (**a**) Drug-treated cells are fixed, permeabilized, deposited in a multi-well glass chamber, and labeled with primary antibodies, and multicolor secondary antibody-QD probes. (**b**) 3D multichannel z-stack images are acquired. (**c**) Discrete QD-tagged protein complexes are counted from image stacks and tabulated for individual cells. (**d**) Single cell phosphoprofiling showing CD34 and pSTAT5 staining. Bee swarm plots depict the phosphoactivity (# of QDs/cell, x axis) for untreated cells and drug-treated cells.

The single cell quantum dot platform (SC-QDP) is a microscopy imaging-based platform that implements molecular quantification of protein levels by counting discrete complexes of proteins in single cells. Cells are drug-treated, fixed, permeabilized, deposited into multi-well chambers, and labeled sequentially with primary phosphoantibodies and secondary antibody-QDs (Fig. 1a). This sequential labeling scheme allows the flexible pairing of any QD color with a phosphoprotein target. Moreover, the characteristic narrow fluorescence emission spectra of QDs allow for ease of QD multiplexing and simultaneous detection of single cell phosphoactivity with other cellular markers (e.g., nucleus, CD34+). The SC-QDP has very high post-assay cell retention and therefore can assay small number of cells (>95%; 250–128,000 cells/well; Supplementary Fig. 1), thus overcoming constraints in the screening of limited sample sizes of primary cells from patients. Multi-channel, z-stack images of phosphoantibody-QD-labeled cells are acquired (Fig. 1b). Automated algorithms count discrete fluorescent complexes of protein molecules in single cells and single-cell phosphoactivity is quantified as the number of discrete QD-tagged phosphoprotein complexes in each cell (Fig. 1c). Cellular debris and cell aggregates are automatically removed and each cell and cell aggregates are automatically removed, and each cell can be viewed to confirm measurements are made in intact single cells. One-dimensional ‘bee swarm’ scatter plots depict the phosphoactivity level for single cells sampled from the total cell population (Fig. 1d).

### Validation of digitized nanoparticle tagged-protein counting by the SC-QDP platform

**Figure 2:**
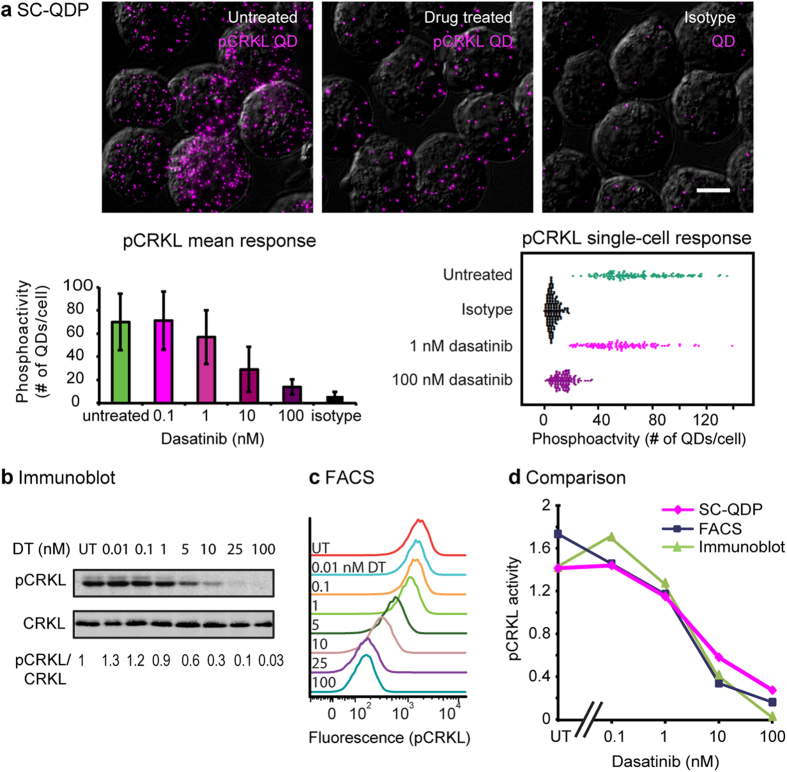
Validation of the SC-QDP approach by immunoblot and FACS. CML K562 cells were processed by SC-QDP, immunoblotting, and FACS assays. pCRKL data are shown; pSTAT5 and pSTAT3 data shown in Supplementary Fig. 2. (**a**) Micrographs of K562 cells processed by SC-QDP for pCRKL in three conditions: untreated, dasatinib-treated (100 nM, 4 h), and no primary antibody (control). Images are collapsed z-stack overlays of pCRKL-QD (magenta) and brightfield DIC channels. Scale bar is 10 μm. Bar graphs show the mean pCRKL activity (y axis), computed as the average number of discrete QD counts in single K562 cells at various dasatinib concentration (x axis). Error bars are standard deviation of the mean. Numbers of cells sampled are: 142, 159, 130, 117, 130, 181, left to right on bar graph. Bee swarm plots show phosphoactivity (# of QDs per cell, x-axis) of cells for conditions of untreated, dasatinib-treated, and no primary antibody control. (**b**) Immunoblot showing pCRKL and CRKL levels in K562 cells treated with increasing concentrations of dasatinib. Quantitative pCRKL/CRKL ratios are indicated below the blots. UT = untreated. (**c**) FACS histograms show pCRKL levels in K562 cells treated with increasing concentrations of dasatinib (DT). UT = untreated. FACS curves at mean ± std: 1,574 ± 971, 1,137 ± 788, 170 ± 115, respectively for untreated, 1 nM, 100 nM conditions. (**d**) Plots compare phosphoactivity (y axis) measured by SC-QDP (blue), FACS (magenta), and quantitation of pCRKL/CRKL from immunoblots (green). Phosphoactivity value at each point is normalized to the mean of the values over the range of drug treatment. Additional validations for pSTAT5 and pSTAT3 are shown in Supplementary Fig. 2.

To validate the digitized nanoparticle-based protein counting approach, we compared SC-QDP, immunoblotting, and FACS assays. We measured pCRKL, pSTAT5, and pSTAT3, surrogate phosphomarkers of the BCR-ABL1, a kinase that is constitutively activated in human K562 chronic myelogenous leukemia cells. Untreated CML K562 cells contain higher levels of QD-pCRKL compared to cells treated with dasatinib kinase inhibitor (KI) treatment (Fig. 2a). Negative control experiments omitting the primary phosphoantibody showed few QD-pCRKL probes per single cell, indicating low non-specific binding of QD-pCRKL probes (control, Fig. 2a). Counts of QD-tagged phosphoprotein complexes for single cells indicate a decrease in mean phosphoactivity level as well as decrease in variance of phosphoactivity with increasing concentration of dasatinib KI (pCRKL, Fig. 2a; pSTAT5, pSTAT3, Supplementary Fig. 2a). The compiled SC-QDP quantitation data also showed low background noise, as indicated by negligible numbers of QDs per cell in the presence of dasatinib (100 nM), which largely suppresses phosphoactivity, and as indicated by controls performed with omission of the primary phosphoantibody (6–7 QDs/cell; Fig. 2a and Supplementary Fig. 2a). Bee swarm scatter plots show the heterogeneity of single cell phosphoactivity. Untreated cells span a wide range of phosphoactivity levels indicating that CML K562 cells exhibit considerable phosphoheterogeneity (pCRKL, pSTAT5, and pSTAT3; Fig. 2a and Supplementary Fig. 2a). Variations in cell division contributed to this heterogeneity, as lovastatin-induced cell cycle synchronization reduced this heterogeneity (data not shown). With KI treatment at higher dose, cellular phosphoactivity shifts to the left, indicating phosphoinhibition, and with a narrowed span, indicating decreased phosphoheterogeneity.

SC-QDP, immunoblotting and FACS analyses were performed in parallel on the same cell samples. Immunoblots showed decreasing phosphoactivity with increasing KI dose for all three phosphoproteins (pCRKL, Fig. 2b; pSTAT5 and pSTAT3, Supplementary Fig. 2b). Similarly, FACS analysis showed that with increasing KI dose, that the peaks of each FACS curve shift to the left, indicating lower phosphoactivity levels, and that the width of each FACS curve narrowing, indicating decrease in phosphoheterogeneity (pCRKL, Fig. 2c; pSTAT5 and pSTAT3, Supplementary Fig. 2c). Quantitative plots of the mean phospholevels as a function of KI dose showed correspondence among all three assays (pCRKL, Fig. 2c; pSTAT5 and pSTAT3, Supplementary Fig. 2c). These plots also captured the steep decline in pSTAT5 levels (Fig. 2b) and more gradual decline in pSTAT3 and pCRKL phosphoinhibition, as a function of dasatinib dose (Supplementary Fig. 2d). Altogether, these data support the validity of the digitized nanoparticle-protein counting approach for quantifying phosphoprotein activity.

### Digitized counting provides ultrasensitive phosphoprotein quantitation

**Figure 3:**
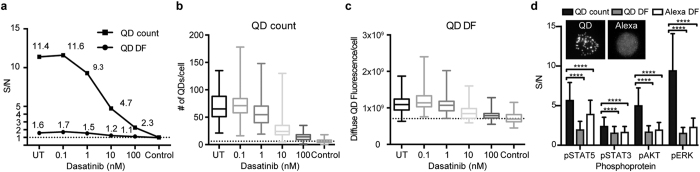
SC-QDP digitized counting sensitivity supersedes conventional summing of diffuse fluorescence in single cells. (**a**) Plot of signal-to-noise (S/N) ratio for pCRKL quantification in K562 cells comparing the SC-QDP method of QD-nanoparticle counting (QD count) to QD diffuse fluorescence (QD DF) averaging in single cells, at increasing dasatinib concentrations. S/N is calculated by dividing the pCRKL level to that of the isotype control. UT is untreated cells. Dashed line is isotype control value. Numbers of cells sampled: 142, 159, 130, 117, 130, and 181 (left to right, x-axis). (**b**) Box plots showing the numbers of QDs/per cell in the SC-QDP from which S/N ratios were computed in Fig. 3a. Dashed line represents the noise which is the QD count for the isotype control. (**c**) Box plots showing the QD-DF per cell for a range of dasatinib concentrations. Dashed line represents the noise, which is the QD DF for the isotype control. Numbers of K562 cells sampled are same as given in panel **a**. (**d**) Single-cell phosphoquantification using the SC-QDP method of single cell QD-probe counting produces superior detection sensitivity compared to QD DF and Alexa DF per cell. Phosphoactivity levels (y-axis) computed in single untreated K562 cells for pSTAT5, pSTAT3, pERK, and pAKT. S/N ratio calculated by normalizing the phosphoactivity levels in untreated cells to the isotype control. Error bars are standard deviation. P values are calculated by the Holm-Sidak multiple comparison test, asterisks denote p value ≤0.0001. Inset shows representative images of pCRKL labeling by QD655 and Alexa 488 reporters in untreated K562 cells. The same primary phosphoantibody used for QD and Alexa labeling. Numbers of cells sampled are n = 637 (+/−169) for QD-labeling, and n = 940 (+/−118) for the Alexa 488- labeling.

We compared digitized counting of discrete QD-tagged phosphoproteins to the conventional method of summing the diffuse fluorescence (e.g. FACS, standard immunocytochemistry) in single cells. Digitized protein quantitation provided markedly higher S/N ratios compared to phosphoquantitation of diffuse QD fluorescence for the same single cell image data (Fig. 3a). Because digitized detection of phosphoprotein levels relies on the number of QDs, whereas diffuse fluorescence quantification can include other fluorescent sources (e.g. cellular autofluorescence), the digitized phosphoprotein signal is better discriminated from the background noise (dotted line, Fig. 3b,c). This improved detection is particularly valuable at high doses of KI in which cellular phosphoprotein content is further decreased (Fig. 3a–c). The signal over the background achieved by SC-QDP counting surpasses that of quantitating the diffuse fluorescence of QD and Alexa fluorescent reporters. This holds true for other phosphoprotein targets (Fig. 3d). The degree of this S/N improvement will depend on the level of abundance of the target phosphoprotein of interest as well as the different binding affinities of the primary antibody. In these experiments, care was taken to use the same primary phosphoantibodies for both QD and Alexa dye labeling, and to optimize the concentrations of each primary phosphoantibody separately for QD and Alexa assay conditions. All together, these data show that digitized nanoparticle-protein counting provides significant improvement that is substantial in detecting differences in important conditions of decreasing in protein abundance, such as by kinase inhibition.

### SC-QDP capture of KI-induced phosphoheterogeneity and insensitivity in single cells

**Figure 4:**
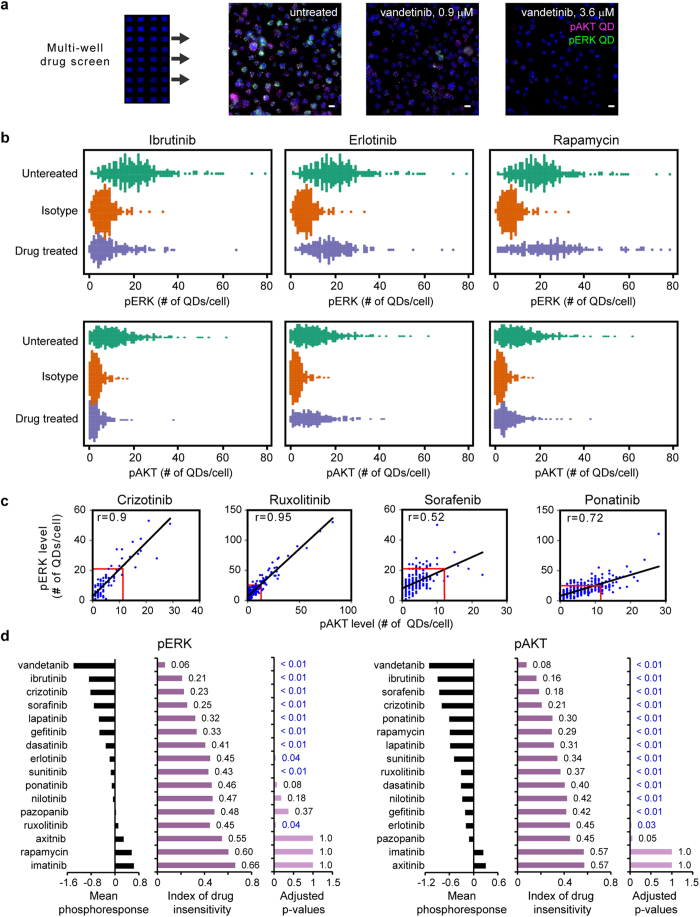
SC-QDP capture of KI-induced phosphoheterogeneity and insensitivity in single cells. (**a**) Multi-well, high-throughput SC-QDP KI screen on MOLM-14 AML cells performed at the IC50 dose for cell death, respectively for each drug (blue, Nuclear Mask). Adjacent images show cells labeled with pAKT-QD (magenta), pERK-QD (green), and Nuclear Mask for untreated and vandetinib treated cells. Scale bar, 10 μm. (**b**) Bee swarm plots show pERK and pAKT levels measured simultaneously in single cells before and after ibrutinib, erlotinib, and rapamycin treatment. Additional plots for vandetanib, axitinib, and imatinib are given in Supplementary Fig. 3. (**c**) Example plots of pERK vs. pAKT levels measured in single cells illustrate a span of correlation values for different kinase inhibitors. Outlier cells exist with pERK and pAKT values equal to or greater than the mean pERK and pAKT values of untreated cells (blue dots outside red rectangles in the plots). (**d**) Compilation of degree of cellular drug insensitivity for the kinase inhibitor panel at the IC50 dose that 50% cell death for each drug. Each drug elicits varying degrees of mean phosphoinhibition in the drug-treated cell population. Mean phosphoresponse is calculated as the difference between the inhibitor-treated mean and the untreated mean, divided by units of standard deviation (σ) of the untreated cells. Scores of phosphoresponse insensitivity are given by the Wilcoxon rank sum test (see Methods). An index of 0.5 indicates untreated and treated cell populations are similar; an index < 0.5 indicates the likelihood that a cell from the drug-treated population has a higher level of phosphoactivity than a cell from the un-treated population. Adjusted p values, blue font shows values where p < 0.05 that indicates a significant difference between untreated and drug-treated cell populations. Number of cells sampled is n = 205 (+/−54) per condition.

We applied the SC-QDP to assess the phosphoresponse of single acute myeloid leukemia (AML)-derived MOLM-14 cells treated with a panel of 16 FDA-approved KIs (Fig. 4a). Bee swarm scatter plots show that the SC-QDP captures differing degrees of KI-induced phosphinihibition and single cell phosphoheterogeneity (Fig. 4b). For example, ibrutinib induced marked pERK and pAKT phosphoinhibition in the overall total cell population but there remain individual cells whose pERK levels are similar to that of the untreated cell population (Fig. 4b). Erlotinib produced a less pronounced inhibition of pERK and pAKT compared to ibrutinib but there also remain insensitive cells with phospholevels similar to that of cells in the untreated population. Rapamycin induced strong pAKT inhibition and less pERK inhibition in the total cell population; however, there in the rapamycin-treated cell population still remain cells with pAKT levels similar to that of the untreated cell population. These, and additional data (Supplementary Fig. 3), demonstrate the SC-QDP capability to capture single cell phosphoheterogeneity under different KI-treatment conditions.

We examined the cells that are less sensitive to KI treatment (Fig. 3b). Outlier cells had high pAKT and/or pERK levels equal to or greater than the mean pERK and pAKT values of untreated cells (cells outside red box, Fig. 4c, data from experiment Fig. 4a). Many outlier cells showed correlated pAKT and pERK insensitivity (crizotinib, ruxolitinib; Fig. 4c), indicating that these cells were KI-insensitive in a dependent manner between pAKT and pERK pathways. In instances of other KIs, outlier cells showed less correlated pERK and pAKT insensitivity (ponatinib, sorafenib; Fig. 4c), indicating KI-insensitivity in one pathway is independent of the other in these cells (e.g. ponatinib, sorafenib, plot, Fig. 4c).

KIs were scored by the probability of finding insensitive cells following drug treatment (Fig. 4d) and defined by an index of drug insensitivity. A Wilcoxson rank sum test was employed to numerically calculate the likelihood of finding cells from KI-treated populations that have higher phosphoactivity than in cells from the untreated populations (Fig. 4d). Merits of the Wilcoxson scoring approach are that it is independent of any assumptions of the nature of the cell population data (e.g. Gaussian distribution) and that it is not based on (arbitrary) threshold selection (see Methods). Indices of insensitivity that equal 50% indicate the highest levels of drug insensitivity, as drug-treated and untreated cell populations are identical. Scores of decreasing probability (<50%) indicate the decreased likelihood of finding insensitive cells in KI-treated conditions; that is, cells that have the same phosphoactivity levels as that found in untreated conditions. The charts show that different KIs in the panel exerted varying degrees of mean pAKT and pERK phosphoresponses (Fig. 4d). As expected, KIs that induce increasingly mean phosphoinhibition response correspond to a decreased likelihood of finding KI-insensitive cells. The SC-QDP captures the wide-ranging levels of cells that remain insensitive following drug-treatment (21–45% for pERK, and 16–45% for pAKT, Fig. 4d). Calculated p-values provide additional confirmation that drug-treated cell populations are statistically different from the untreated cell population (blue font, p<0.05, right hand bar plot, Fig. 4d). These data show that the SC-QDP approach enables assessment of the potency of a KI not only by its mean phosphoinhibition level, but also by the completeness of its inhibitory effect in the entire cell population.

### SC-QDP identifies KI- insensitive CD34+ cells in chronic myeloid leukemia patients

**Figure 5:**
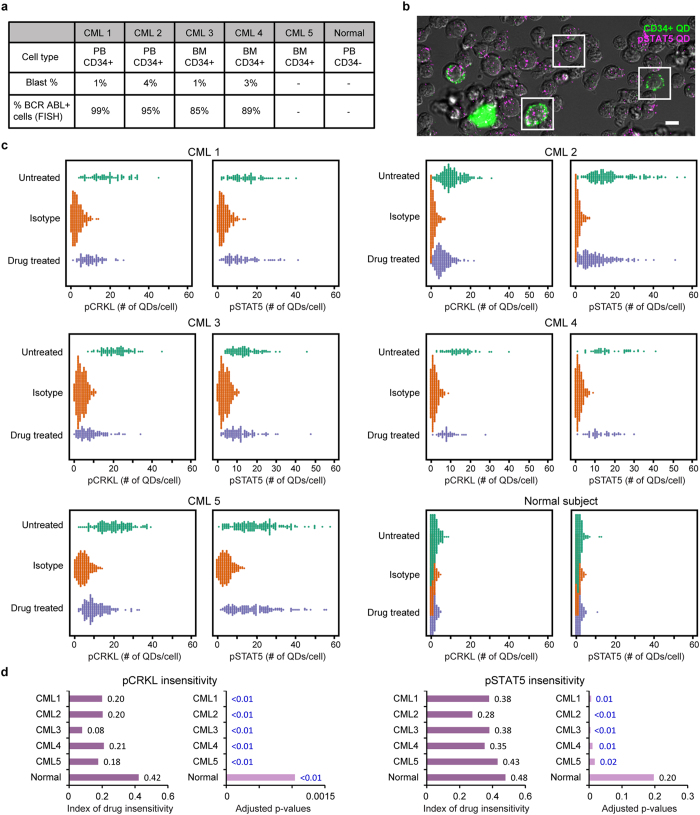
SC-QDP identifies dasatinib kinase inhibitor-insensitive CD34+ cells in newly diagnosed CML patients. (**a**) Cell type, blast percentage and BCR-ABL1 positivity for CML specimens. PB = peripheral blood, BM = bone marrow, dashes = unavailable/not applicable data. (**b**) QD-labeled CML patient cells show heterogeneity in CD34 positivity (green) and pSTAT5 (magenta) expression. Framed areas show representative CD34+ (green) and CD34− cells, with varying numbers of pSTAT5-QD probes in each cell (magenta). Scale bar = 10 μm. (**c**) Bee swarm scatter plots of pCRKL and pSTAT5 profiles of CD34+ cells from CML patients and CD34− cells from a healthy subject. Cells were treated with 100 nM dasatinib for 4 h. Scored values of phosphoresponse insensitivity are given by the Wilcoxon rank sum test (see Methods). An index of insensitivity of 0.5 yields the highest level of insensitivity as untreated and treated cell populations are equal; an index that approaches zero indicates the lower likelihood of insensitive cells. Adjusted p-values indicate a significant difference between untreated and drug-treated cell populations (blue text indicates values of p < 0.05). The number of CD34+ cells sampled for pCRKL activity for the five patients was: 80, 87; 195, 196; 160, 191; 91,108 and 56, 45 for untreated and inhibitor-treated conditions, respectively. CD34+ cells sampled for the measurement of pSTAT5 activity for the five CML patients was n = 82, 91; 179, 190; 177, 159; 100, 112 and 47, 47 for untreated and inhibitor-treated conditions, respectively. The healthy subject stained negative for CD34+ cells; the number of CD34- MNCs sampled from the healthy subject for pCRKL measurements was n = 274; 216 for untreated and inhibitor-treated conditions, respectively; and for pSTAT5 measurements was n = 268; 226 for untreated and inhibitor-treated conditions, respectively.

The identification of kinase inhibitor insensitivity in rare primitive cell populations from primary leukemia patient samples by direct phosphoresponse measurement has been challenging to routinely accomplish using standard immunoblotting and FACS methods^14^. We tested the detection capability of the SC-QDP for identifying rare kinase inhibitor insensitive cells by assaying single CD34+ cells obtained from patients with chronic myelogenous leukemia (CML). SC-QDP analysis was performed on peripheral and bone marrow-derived mononuclear cells (MNCs) from 5 patients with newly diagnosed CML (Fig. 5a). These patients had high leukemic cell burden; FISH cytogenetics showed that 85–99% of undifferentiated blood cells (blasts) were positive for BCR-ABL1, the chromosomal abnormality that underlies most CML. These patients also exhibited low blast levels (1–4%, Fig. 5a) that is consistent with early stages of CML. MNCs from these patients were treated with the potent BCR-ABL1 kinase inhibitor, dasatinib (100 nM, 4 h). SC-QDP identified single CD34+ cells, a marker of undifferentiated leukemic cells, in these patients, whereas, as expected, a healthy control subject was negative for CD34+ cells (Fig. 5a). SC-QDP measured the levels of pCRKL and pSTAT5, two surrogate markers of overactive BCR-ABL1 signaling, in single CD34+ cells in 5 new CML patients^10^,^24^,^25^.

The SC-QDP identified subpopulations of single-cell MNCs with CD34 positivity and quantified phosphoactivity in CML patients. Bee swarm plots show that at baseline, there exists a wide range of heterogeneity of pCRKL and pSTAT5 in single CD34+ cells (Fig. 5c). Dasatinib treatment induced overall pCRKL inhibition; however, in all 5 patients, there remain insensitive cells with pCRKL levels that were similar to that of the untreated cells (Fig. 5c). Numerical scores obtained from Wilcoxson rank sum test showed that the CML3 patient had effective treatment whereas the other 4 patients showed a ~20% probability that there remain cells with pCRKL levels similar to cells in untreated cell populations (Fig. 5d). P-value calculations show that treated and untreated cell populations are significantly different from one another (blue text, Fig. 5d). SC-QDP analysis performed on the healthy control subject’s CD34− cells showed that pCRKL levels were low in both dasatinib-treated and untreated conditions and comparable to the noise level of the control isotype (Fig. 5d). Adjusted p-value tests show that while dasatinib-treated vs untreated cell populations derive from statistically different distributions (blue text, adjusted p-value plots for p< 0.05, Fig. 5c), the likelihood of finding cells with pCRKL similar to that of untreated populations was close to 50%, indicating both distributions are similar. These data demonstrate the ultrasensitive ability of the SC-QDP to clearly identify dasatinib- insensitive CD34+ cells within leukemia patient samples.

SC-QDP’s readily expandable panel of markers facilitated identification of insensitive cells in these same five patients using pSTAT5, a surrogate marker of BCR-ABL1 activity that is further downstream of pCRKL^10^,^24^,^25^. With the exception of the CML5 patient who showed dasatinib-induced inhibition (index score = 28%), dasatinib treatment provided only mild reduction of pSTAT5 levels (index score = 38–43%) (Fig. 5c). This higher phosphoinsensitivity of pSTAT as compared to pCRKL suggests that other signaling pathways could compensate for STAT5 phosphorylation^25^. As expected, pSTAT5 levels measured in MNCs from a healthy subject were close to assay noise in dasatinib-treated, untreated, and isotype control conditions with an index score of 48%, indicating similar distribution in this patient of dasatib-treated and untreated cell populations (Fig. 5c).

Overall, these data demonstrate the capability of the SC-QDP to sensitively identify rare subpopulations of cells that are insensitive to kinase inhibitors in primary leukemia patients. Other methods have not provided such direct phosphorylation-based identification of cellular phosphoinsensitivity^12^,^14^.

## Discussion

Many important proteins are expressed in low abundance and therefore difficult to detect and quantitate in single cells. We demonstrate a digitized molecular counting approach that addresses limitations in attaining sufficient sensitivity necessary to reliably detect and quantify levels of activated proteins above the background noise. By counting discrete fluorescent nanoparticle-tagged protein complexes, we show that this digitized molecular counting approach achieves a detection sensitivity in single cells that supersedes conventional fluorescence averaging measurements (Fig. 3). Digitized quantitation detects discrete fluorescent signal and therefore is less susceptible to background noise that may arise from diffuse fluorescent sources (e.g. cellular autofluorescence). Given that many proteins of interest and disease importance, such as signaling and regulatory proteins, are present at low abundance in single cells^3^,^4^,^5^,^26^, and that such proteins can be further reduced by treatment with therapeutic compounds, this digitized molecular counting approach is of broad value for the quantitative study of major cellular signaling processes, as well as to detect other molecules of expected low abundance (e.g. ligands, drugs, viral particles) in single or rare populations of cells.

The value of the high sensitivity achieved by digitized proteomic quantitation is illustrated by our implementation of an integrated SC-QDP platform to measure phosphoprotein signaling in individual leukemia cells. SC-QDP enabled identification of single or small number of outlier cells with high pAKT and/or pERK insensitivity to a number of KI drugs in current clinical use. SC-QDP also provided metrics for assessing KIs based on single cell quantitative phosphomeasurements. This, in turn, showed the diverse and complex dose-dependent modulation of different kinase inhibitors on phosphotargets, including some behaviors that are not necessarily expected for classic phosphoinhibition, and enabled assessment of KI potency by the completeness of its inhibitory effect (e.g. KI- drug insensitivity) in entire sampled cell populations. In addition to drug-screening capabilities, the value of molecular-sensitive SC-QDP measurements was illustrated in clinical samples by identification of CD34+ cells that are phosphoinsensitive to the kinase inhibitor, dasatinib, the standard KI for many leukemia patients with CML. While the existence of dasatinib-insensitive cells has been identified by genomic and cell proliferation studies^14^,^20^, technical limitations in immunoblotting and FACS methods have impeded the direct identification of phosphoactivity in rare subpopulations of dasatinib-insensitive cells^14^. Our findings of dasatinib-insensitive CD34+ cells (Fig. 5c and Supplementary Fig. 4), in the CML patients sampled point to incomplete inhibition of BCR-ABL gene, or downstream phosphoprotein target activity as an underlying mechanism in CML persistence. Given that protein phosphorylation is a key regulator of fundamental cell signaling processes and aberrant protein phosphorylation is implicated in hundreds of human diseases, including cancer and neurodegenerative disorders^27^, the highly sensitive measurement of phosphotarget inhibition is of value for uncovering mechanisms that may contribute to cellular heterogeneity in disease persistence^28^,^29^,^30^ as well as for identification for aberrant signaling in rare outlier cell subpopulations that may be important for guiding drug selection in individuals.

Our study suggests that ultrasensitivity afforded by digitized quantitation may be a broadly useful approach to protein quantitation. Examples of discrete counting of RNA has revealed variation in expression levels for genes amongst cells^31^ and a recent study employed proteomic quantitation in single cells using counts of fluorescent bar-coded reporters^32^ however, a direct comparison of discrete molecular counting versus diffuse quantitation of proteins or other molecules has not, to our knowledge, been reported. Digitized protein quantitation can be implemented in different formats in single cells or tissue. Labeling of cell or tissue by discrete probes need not be limited to quantum dots, but other bright discrete imaging probes, using existing software packages such as ImageJ and Cell Profiler to perform cell segmentation and particle counting. Our specific implementation of the SC-QDP incorporates several additional advantageous features to apply the power of digital protein counting. The microscope-based platform of the SC-QDP offers the capability to better visually discern single cells from artifact (e.g. debris, aggregates), which is practically useful for accurate identification of rare cells. Another valuable feature of the SC-QDP platform is its capability to accommodate smaller samples of limited cell number with minimal cell loss (<5% cell loss at 250–128,000 cells, compared to 46–75% cell loss in FACS (Supplementary Fig. 1). This overcomes severe bottlenecks that currently exist in multi-drug/combinatorial proteomic profiling in samples using limited primary patient materials.

In summary, digitized protein quantitation and its implementation by SC-QDP are powerful proteomic tools for evaluating drug response and developing new treatment strategies for protein targets that may be difficult to reliably detect and quantitate in single cells. In this emerging era of precision medicine, such tools, when coupled with other proteomic and next-generation, single-cell sequencing approaches^33^,^34^,^35^, can help to provide a more complete picture of disease and effective remedies.

## Methods

### Cell culture and kinase inhibitor treatment

Primary mononuclear cells (MNCs) were isolated from peripheral blood or bone marrow of newly diagnosed, untreated CML patients. Study protocols for using human cells were reviewed and approved by the institutional review board of Oregon Health & Science University. All the methods were carried out in accordance with the approved IRB guidelines (IRB# 4422). All patients signed an IRB-approved informed consent prior to study participation. MNCs isolated by Ficoll-Hypaque (Sigma-Aldrich) density gradient centrifugation and cultured in serum-free media consisting of IMDM supplemented with 20% BIT (Stem Cell Technologies), 40 μg/ml low-density lipoprotein (Sigma-Aldrich), 10^–6^ M β-mercaptoethanol, and the following cytokines: 100 ng/ml SCF (Stem Cell Technologies), 100 ng/ml G-CSF, 20 ng/ml FLT3 ligand, 20 ng/ml IL-3, and 20 ng/ml IL-6 (Sigma-Aldrich). MNCs were grown at a density of 5 × 10^5^ cells/ml at 37 °C, 5% CO_2_, overnight in a humidified incubator before drug treatment. The CML (K562) and AML (MOLM-14) cell lines were grown in suspension culture, in RPMI with 10% fetal bovine serum (Invitrogen), 1% L-glutamine (100 mM), and 1% penicillin/streptomycin (100 μg/ml), in a humidified incubator at 37 °C, 5% CO_2_.

K562 cells and MNCs were treated for 4 h with dasatinib, at a concentration of 100 nM unless otherwise specified. For the multipanel kinase inhibitor screening, MOLM-14 cells were treated for 48 h with a panel of FDA-approved kinase inhibitors. Drug doses were equivalent to the IC50 drug dose that elicits 50% cell death (drug concentrations listed in Supplementary Methods). IC50 cell death values were obtained by kinase panel screening performed on the same MOLM-14 cell lines by MTS assay (CellTiter 96-Aqueous One Solution, Promega); as previously described^36^.

### Immunoblotting analysis

K562 cell lysate was prepared by boiling cells in a SDS-PAGE loading buffer. Equivalents of 5 × 10^5^ cells per untreated or dasatinib-treated conditions were subjected to SDS-PAGE and were immunoblotted with primary antibodies; pCRKL, CRKL, pSTAT5, STAT5, pSTAT3 and STAT3 (see Supplementary Methods for details). Phosphoprotein signal was imaged on a Lumi Imager.

### FACS analysis

Untreated and kinase inhibitor-treated K562 cells were fixed and permeabilized according to the manufacturer’s instructions (BD Biosciences); incubated with antibodies: pCRKL, pSTAT3 or pERK (see Supplementary Methods for details); and analyzed on a FACS Aria instrument (BD Biosciences).

### SC-QDP phosphoprotein labeling

Cells were fixed, permeabilized and dispersed onto multiwell cover glass chambers (Lab-Tek; Nunc, or custom-made). The cells were blocked and treated with primary anti-phosphoprotein antibodies: pCRKL, pSTAT5, pSTAT3 or pERK1/2 (see Supplementary Methods for details). The anti-CD34 was used for selection of CD34+ primitive cells. Following primary antibody incubation for 2 h, cells were treated with secondary anti-IgG-QD or anti-IgG-Alexa 488 antibodies (Life Technologies).

### SC-QDP image acquisition

Cells were imaged with an inverted fluorescent microscope (Zeiss AxioObserver) equipped with high magnification objectives, QD filter sets (Semrock), and a Luca-R camera (Andor) suitable for detecting discrete QD fluorescence. Data acquisition consisted of acquiring multiple fields of view randomly for each well of the multi-well chamber. The microscope was controlled by Micromanager (https://www.micro-manager.org/)^37^. For each field of view, z-stacks were acquired in appropriate QD fluorescent channels (inter-slice distances of 275–300 nm, total z-depth of 18 μm for K562 cells, and 10–12 μm for MOLM-14 cells and MNCs from patients) along with a DIC image at mid-stack. Image acquisition was performed manually or by automated high-throughput scanning. For automated high-throughput scanning, we used a custom-written Java plugin for Micromanager that allows scanning of user-selected wells in multiple channels.

### Discrete SC-QDP counting and diffuse fluorescence quantification in single cells

Phosphoprotein activity levels were quantified in single cells using automated software imaging algorithms. Cell segmentation was first performed manually and then optimized for speed and accuracy by threshold-based object detection in CellProfiler^38^ (see Supplementary Methods for details). Cell outline coordinates were exported for subsequent single cell QD counting in MATLAB software.

Discrete QD fluorescence was detected, localized and tabulated on a per cell basis using automated algorithms written in MATLAB. Detection of discrete QD probes was accomplished using radial symmetry localization^39^ of QDs in single-cell z-stacks (see supplementary methods for details). The QDs were identified as discrete, non-aggregated units comprised of single or a few QDs, as confirmed by intensity profile measurements of QD fluorescence^40^,^41^.

Measurements of single-cell total diffuse fluorescence of QD and Alexa dyes were obtained for each cell by summing pixel values from all slices in a single z-stack corresponding to each cell and subtracting the global background value for each field of view from the image. The global background value was computed as the mean of the minimum pixel value corresponding to each y-column of the entire field of view in each image.

### SC-QDP single-cell phosphoresponse profiles and scoring of drug insensitivity

We use the Wilcoxon rank-sum test (also known as the Wilcoxon-Mann-Whitney test or the Mann-Whitney U test^42^,^43^, to make statistical comparisons between the populations of untreated and drug-treated cells. This non-parametric test is robust in that it does not rely on the type of distribution of underlying cellular populations (e.g. assumption of normal distributions such as for the *t*-test). We apply the Wilcoxon rank-sum test to determine whether two populations (X, untreated cells and Y, drug-treated cells) have similar distributions or, as an alternative hypothesis, whether one population tends to have significantly lower values of phosphoactivity than the other. The null hypothesis (H0) is that X (untreated) and Y (drug-treated) have similar distributions. If the null hypothesis is true, then probability that an observation from X is less than an observation from the Y equals the probability of an observation from Y is less than an observation from X, which can be represented as: *P*(*X* < *Y*) = *P(X* > *Y*) or *P*(*X* < *Y*) +0.5P(*X* = *Y*) = 0.5. We approximate these probabilities using the samples we have (i.e., QD counts in cells from both populations). We define an ‘*index of drug insensitivity*’ as: *P*(*X* < *Y*) +0.5*P*(*X* = *Y*), which compares the QD counts of cell pairs from X and Y and gives the probability that the cell in Y has a higher QD count than the cell in the X for a random pair of cells. Let *X* = {*x*_1_, *x*_2_, …, *x*_M_} and *Y* = {*y*_1_, *y*_2_, …, *y*_N_} be the number of QD counts of cells in untreated and drug-treated cell populations, respectively, where *M* and *N* are the numbers of cells in these populations. The *index of drug insensitivity* can be approximated by:


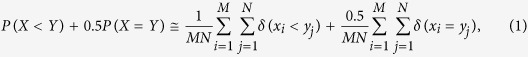


where *δ* represents the Kronecker delta function that returns 1 if its argument is true and 0 otherwise. If drug treatment is ineffective, then X and Y will have similar distributions, P(X > Y) = P(X < Y), and the index score should be close to 0.5. If drug treatment is effective, then the Y population should have significantly lower values of phosphoactivity, and the index score should be smaller than 0.5.

We calculate adjusted p-values to provide a measure of confidence that there is a statistically significant difference between population X and Y. The adjusted p-values provide statistically more meaningful p-values that account for hypothesis testing of multiple drugs as a group. Thus, if p < 0.05, we can conclude that the populations X and Y are statistically different and that we have high confidence that the score obtained from index of drug insensitivity is statistically significant. If p > 0.05, then we can not reject the null-hypothesis that the two populations may be from the same population even if the index of drug insensitivity may be different than 0.5.

## Additional Information

**How to cite this article**: Jacob, T. *et al*. Ultrasensitive proteomic quantitation of cellular signaling by digitized nanoparticle-protein counting. *Sci. Rep.* **6**, 28163; doi: 10.1038/srep28163 (2016).

## Supplementary Material

Supplementary Information
