# Supplementary material for: Ultrasensitive proteomic quantitation of cellular signaling by digitized nanoparticle-protein counting
Source: Sci Rep. 2016 Jun 20;6:28163. doi: 10.1038/srep28163 (PMC4913309; doi:10.1038/srep28163)
Supplement: Supplementary Information [file srep28163-s1.pdf]

Ultrasensitive proteomic quantitation of cellular signaling by digitized nanoparticle-protein counting

Thomas Jacob, Anupriya Agarwal, Damien Ramunno-Johnson, Thomas O'Hare, Mehmet Gönen, Jeffrey W. Tyner, Brian J. Druker, Tania Q. Vu

Supplementary Information

Supplementary Methods

Supplementary Figure 1. High cellular recovery from the SC-QDP multi-well chamber enables handling of samples of limited numbers of cells.

Supplementary Figure 2. Validation of SC-QDP by immunoblotting and FACS analyses using additional pSTAT5 and pSTAT3 phosphoprotein targets.

Supplementary Figure 3. Additional single-cell drug response profiles from multi-drug SC-QDP screening illustrating pERK and pAKT heterogeneity and KI-insensitivity.

Supplementary Methods

Antibodies used for Immunoblot analysis are anti-pCRKL (Tyr207) #3181, CRKL, (clone 32H4), #3182, anti-pSTAT5 (Tyr694), (clone C11C5) #9359, STAT5 (#9363) anti-pSTAT3 (Tyr705)#9131, STAT3, (clone 124H6), #9139 antibodies (Cell Signaling Technology) at 1:1000 in TBST overnight, after being blocked with 5% BSA in TBST. Phosphoprotein signal was imaged on a Lumi Imager.

Antibodies used in FACS analysis: Cells incubated with anti-pCRKL-PE (Tyr207), (clone K30-391.50.80) #560788, anti-pSTAT5-Alexa 488 (Tyr694), (clone 47/Stat5/pY694) #562075, anti-pSTAT3-PE (Tyr705), (clone 4/P-STAT3) #612569, or anti-pERK1/2-Alexa 488 (Thr202/Tyr204), (Clone 20A) # 612592 antibodies (all from BD Biosciences) for 1 hr in the dark; washed twice with PBS supplemented with 1% BSA; and analyzed on a FACS Aria instrument (BD Biosciences).

SC-QDP phosphoprotein labeling. The cells were blocked and treated with primary anti-phosphoprotein antibodies anti-pCRKL (Tyr207) #3181, anti-pSTAT5 (Tyr694) (clone C11C5) #9359, anti-pSTAT3 (Tyr705) #9131, and anti-pERK1/2 (Thr202/Tyr204) (clone E10) #9106 antibodies (Cell Signaling Technology) The anti-CD34 (Qbend-10) antibody (Dako, USA #M7165) was used for selection of CD34+ primitive cells. Following primary antibody incubation for 2 h, cells were treated with secondary anti-IgG-QD or anti-IgG-Alexa 488 antibodies (Life Technologies); anti-mouse IgG-QD605 (#11002MP), anti-rabbit IgG-QD-605 (#11402MP), anti-rabbit IgG-QD-655 (#Q11422MP), anti-mouse IgG-Alexa 488 (#A11029), and anti-rabbit IgG-Alexa 488 (#A21206). Concentrations of primary and secondary antibody probes were optimized for each anti-phosphoprotein marker to yield low background.

FDA-approved kinase inhibitor panel and the concentrations of the drugs: The following concentrations of high and low drug doses were used: axitinib (100 nM, 25 nM), dasatinib (400 nM, 100 nM), erlotinib (7 μM, 1.75 μM), gefitinib (9 μM, 2.25 μM), pazopanib (2 μM, 0.5 μM)), imatinib (10 μM, 2.5 μM), ruxolitinib (10 μM, 2.5 μM), lapatinib (9 μM, 2.25 μM), nilotinib (9 μM, 2.25 μM), ibrutinib (1.6 μM, 0.4 μM), crizotinib (1.8 μM, 0.45 μM), ponatinib (4 nM, 1 nM), rapamycin (20 nM, 5 nM), sorafenib (8 nM, 2 nM), sunitinib (12 nM, 3 nM), and vandetanib (3.6 μM, 0.9 μM).

Cell segmentation by CellProfiler. Fluorescent image of the nucleus stained with HSC NucelarMask Deep Red Stain (Life technologies) was used for identifying the primary object (nucleus). Standard 'analysis modules' in the CellProfiler were selected for nuclei identification. Typical diameters of 50 pixel minimum and 90 pixel maximum were selected for identification of primary objects. The parameters including 'global threshold strategy', 'background thresholding method', and 'automatic smoothening method for thresholding' were selected for primary object identification. A threshold correction of 1.0 was applied. The intensity-based method was applied to distinguish the clumped objects. Primary objects were expanded by 6 pixels using the 'Distance-N' method for identification of the secondary objects (cell outlines). The nuclear and cell outlines were overlaid on the DIC image and the image file saved for visual validation of cell segmentation.

QD detection and counting: The QD localization precision was ~20 nm in the x-y plane using radial symmetry localization. Redundant QDs found in the identical locations in three or fewer consecutive z-slices were considered as a count of one QD. The coordinates of uniquely identified QD were overlaid with the Cell Profiler cell segmentation output file to define the location and total number of QDs for each single cell z-stack. Automated algorithms for counting discrete fluorescent QD-phosphoprotein complexes were validated by comparing to manual counting and showed a maximum difference of 3%. QD counts represent the relative activity levels of a specific phosphoprotein, rather than the absolute count of all phosphoactivated proteins, as antibodies are full-length and may tag >1 activated phosphoprotein molecule.

Supplementary Figure 1. High cellular recovery from the SC-QDP multi-well chamber enables handling of samples of limited numbers of cells.

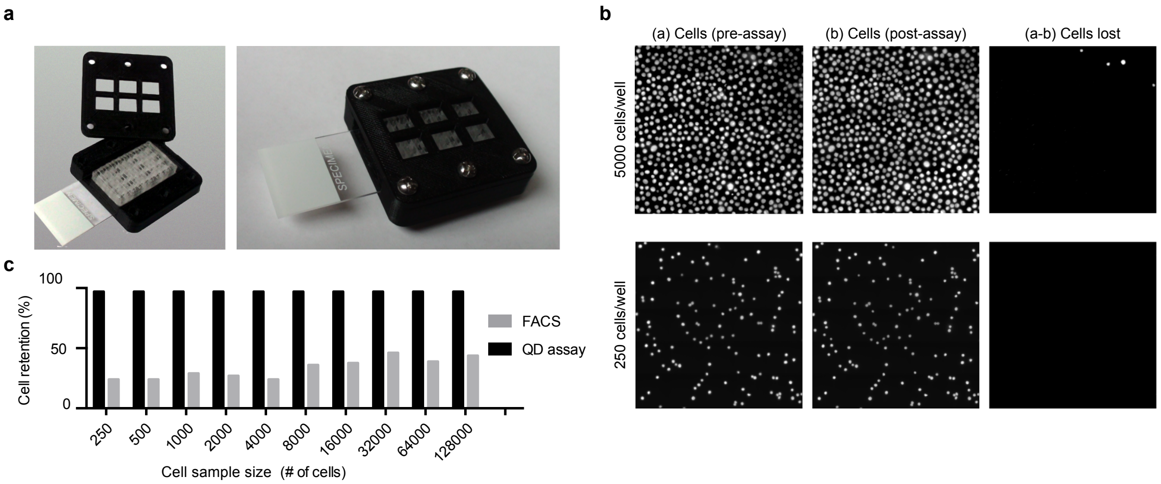

a) Components (left) and the assembly (right) of custom SC-QDP multi-well glass chamber. Multi-well chambers use low volumes of reagent (10-20 μl/well, 36-60 wells) and are used for both phosphoprotein labeling and imaging. **b)** Probe labeling in multi-well chamber shows high cell retention that enables handling of samples with limited numbers of cells. Example micrographs of CML K562 cells before and after SC-QDP probe labeling show very few cells lost after the assay, even for small numbers of cells (250 and 5,000 cells/well). Each micrograph is a composite of 25 fields of view from one well. Cells are labeled with CellMask. **c)** Quantitative comparison of cell retention after SC-QDP and FACS processing of identical CML K562 cell samples shows higher cell retention (>95-99%) with SC-QDP compared to FACS (24%-53%) for a broad range of cell numbers (250-128,000). Plots are the percentage of cells retained as a function of the initial number of cells per sample.

Supplementary Figure 2. Validation of SC-QDP by immunoblotting and FACS analyses using additional pSTAT5 and pSTAT3 phosphoprotein targets

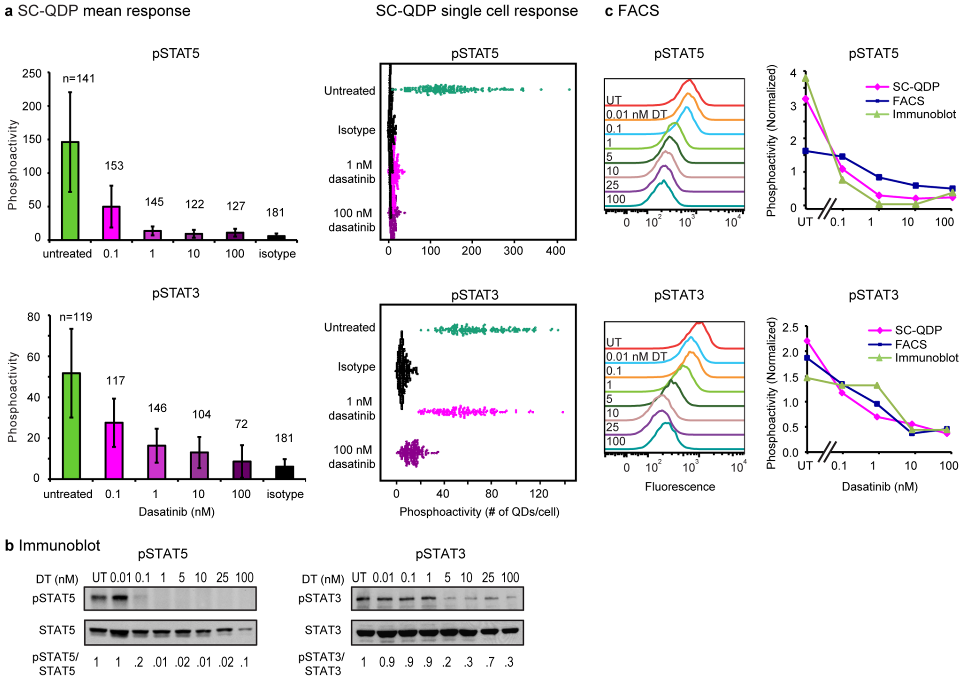

Data corresponds to Figure 2, for two additional phosphoproteins, pSTAT5 and pSTAT3. **a)** Bar graphs (left) show the mean phosphoactivity (y axis), as measured by the average of discrete QD counts, and computed from single K562 cells for each dasatinib drug dose (x axis). Error bars show standard deviation. n is the number of cells sampled. Bee swarm plots show single cell phosphoactivity (# of QDs per cell, x-axis) for untreated, drug-treated,, and control no primary phosphoantibody in K562 CML cells. **b)** Immunoblots show pSTAT5 (Tyr694), (clone C11C5) and total STAT5 and pSTAT3 and total STAT3 (clone 124H6), levels in K562 cells treated with dasatinib at increasing concentrations. Quantitation of pSTAT5 and pSTAT3 are normalized by STAT5 and STAT3 respectively and are indicated below the blots as pSTAT5/STAT5 and pSTAT3/STAT3 ratios. UT = untreated. **c)** FACS histograms (left) show pSTAT5 and pSTAT3 levels in K562 cells treated with dasatinib (DT) in increasing concentrations; UT = untreated. Line plots (right) compare phosphoactivity (y axis) as measured by SC-QDP assays (blue), FACS (magenta) and quantitation of pSTAT5/STAT5 and pSTAT3/STAT3 immunoblot data (green). Phosphoactivity value at each point is normalized to the mean of the values over the range of drug treatment.

Supplementary Figure 3. Additional single-cell drug response profiles from multi-drug SC-QDP screening illustrating pERK and pAKT heterogeneity and KI-insensitivity.

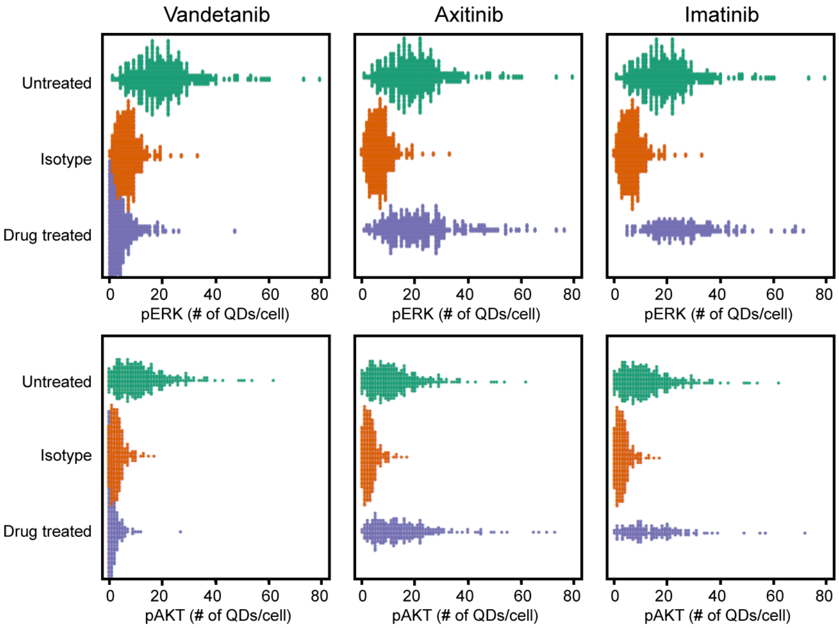

Single cell bee swarm plots of pERK and pAKT activity measured under conditions of untreated, and drug-treatment. Drug doses at IC50 (50% cell kill) for each kinase inhibitor: vandetinib (10 μM), axitinib (0.1 μM) and imatinib (10 μM). n=205 (+/- 54) cells per condition.
